# Do anticoagulants impact the “in-house mortality” after surgical treatment of proximal femoral fractures—a multivariate analysis

**DOI:** 10.1007/s00264-022-05503-0

**Published:** 2022-07-26

**Authors:** Annabel Fenwick, Michael Pfann, Jakob Mayr, Iana Antonovska, Andreas Wiedl, Stefan Nuber, Stefan Förch, Edgar Mayr

**Affiliations:** Department of Trauma, Orthopedic, Plastic and Hand Surgery, University Hospital of Augsburg, Stenglinstrasse 2, 86156 Augsburg, Germany

**Keywords:** Proximal femur fracture, Anticoagulants, Mortality rate, Time to surgery

## Abstract

**Purpose:**

The prevalence of proximal femur fractures is increasing with rising population age. Patients are presenting with more comorbidities. Anticoagulants create a challenge for the necessary early surgical procedure (osteosynthesis or arthroplasty). Our aim was to investigate the influence of anticoagulants on in-house mortality after surgical treatment of proximal femoral fractures.

**Methods:**

A retrospective single-centre study was conducted including 1933 patients with an average age of 79.8 years treated operatively for a proximal femoral fracture between January 2016 and June 2020. One treatment protocol was performed based on type of anticoagulant, surgery, and renal function. Patient data, surgical procedure, time to surgery, complications and mortality were assessed.

**Results:**

On average, patients with anticoagulants had a delay to surgery of 41.37 hours vs 22.1 hours for patients without (*p* < 0.000). Anticoagulants were associated with the occurrence of complications. The total complication rate was 22.4%. Patients with complications showed a prolonged time to surgery in comparison to those without (28.9 h vs 24.9 h; *p* < 0.00). In-house mortality rate was 4% and twice as high for patients on anticoagulants (7.7%; *p* < 0.00). Whilst there was no significant difference in the mortality rate between surgery within 24 and 48 hours (2.9% vs. 3.8%; *p* < 0.535), there was a significant increase in mortality of patients waiting more than 48 hours (9.8%; *p* < 0.001).

**Conclusions:**

Pre-existing anticoagulant therapy in patients with proximal femur fractures is associated with a higher mortality rate, risk of complications and prolonged hospital stay. Further influential factors are age, gender, BMI and time to surgery.

## Introduction

Proximal femoral fractures are amongst the third most common fractures [1, 2]. The prevalence in Germany is around 135,000 per year and rising with an increasingly ageing population [3]. Besides severe impact on daily life, pre-existing mobility, return to daily activities and an extremely high mortality rate of 20 to 30% within the first year, there are also immense economic costs linked to the length of hospital stay, intensive care, rehabilitation and caretaking [4, 5].

More patients are admitted to hospital with comorbidities and multimedication including anticoagulant therapy. In Germany alone, the estimated number of patients with anticoagulants including anti-platelet therapy has risen to about one million patients. Especially direct oral anticoagulants DOACs are increasingly prescribed and there are no current standard protocol or guidelines for peri-operative management in hip fractures. [6]

Early surgery and geriatric co-management have emerged as influencing factors for improved outcome and decreased mortality rates. But nearly all results are based on retrospective data [7–11].

This leads to a variety of problems that have to be considered in the treatment algorithm of proximal femoral fractures. Besides the choice of optimum surgical procedure and timing of surgery also anticoagulants and their influence as well as further outcome determining factors such as mobility, peri-operative complications and demographic data must be considered and put into relation to the accompanying risks.

The following study had the purpose of investigating the most dominant factors influencing in-house mortality with emphasis on anticoagulant therapy after surgical treatment of proximal femoral fractures.

## Material and methods

### Data acquisition

For our retrospective cohort single-centre study (level III), all patients treated operatively for a proximal femoral fracture (femoral neck, pertrochanteric and subtrochanteric fractures) at our level I trauma centre between January 2016 and June 2020 were evaluated. Exclusion criteria were primary conservative treatment, greater trochanteric fractures, periprosthetic fractures and referrals for revision surgery and polytraumatised patients.

The study conducted was approved by the local Ethics Committee and fulfils the standards of the Declaration of Helsinki (20–2155-101).

The charts were reviewed for demographic data such as age, gender, BMI, comorbidities including Charlson Comorbidity Index CCI [12] and ASA American Society of Anaesthesiologists classification [13], fracture morphology, co-geriatric management, medication, especially anticoagulants, mobility pre- and post-operatively, revisions and concomitant fractures. If re-admitted during the above-mentioned period for the contralateral side, patients were included again as a separate case. Type of surgery and time to surgery from admission, length of stay on intensive care unit and overall length of hospital stay (LHS) were analysed. Complications were recorded and divided into urinary infections, pneumonia, embolism or thrombosis, haematoma, wound infections and mechanical complications, i.e. post-operative fracture or dislocation or cutting out. In-house mortality and cause were evaluated.

### Therapy

One consistent therapy protocol was applied throughout the total period reviewed. Target time to surgery was within 24 h of admission for all patients without anticoagulation or only anti-platelet therapy (AP), including dual AP therapy. Patients with DOACs (edoxaban, rivaroxaban, apixaban) were divided into two groups according to their kidney function (Gr 1: GFR > 50, Gr 2: GFR < 50). If renal clearance was good, surgery was performed within 24 h. If renal function was impaired, surgery was postponed to 24–48 h after admission to reduce risk of bleeding. For patients with dabigatran, the time limits were prolonged to 36 and 72 h. All patients with warfarin therapy were closely monitored and supplemented with intravenous Vit K if possible (exceptions: mechanical valve, thrombembolic event within the last three months, congestive heart failure, EF < 20%). Surgery could go ahead when the quick value reached 60%. No switching or bridging was done pre-operatively. PPSB was not administered, as it is still off-label use. Depending on pre-operative mobility, comorbidities and fracture morphology total or hemi-arthroplasty (cemented or uncemented, Fa. Zimmer Biomet) was performed for femoral neck fractures, intramedullary nailing PFNa, Fa. Synthes (± cerclage) for pertrochanteric fractures and plate/screw osteosyntheses (DHS, dynamic hip screw, Fa. Synthes) for undisplaced pertrochanteric or lateral femoral neck fractures. All subtrochanteric fractures were addressed by open reduction, cerclage and cephalomedullary nailing in side positioning. Thirty minutes prior to surgery, all patients received an i.v. single shot of 2 g cefazolin.

Post-operatively, venous thrombembolism prophylaxis was given from day one with enoxaparin 40 mg subcutaneously. Anticoagulants were substituted with tinzaparin sodium according to patient weight post-operatively. All patients were allowed full weight bearing immediately after surgery and received physiotherapy from day one. Labs were taken on the first, fourth to sixth day post-operatively to determine blood loss. Haemoglobin levels under 7 g/l received blood transfusions if consented. Between 7 and 8 g/l transfusions were done depending on symptoms and cardiovascular risk factors.

### Statistical analysis

Statistical analysis was carried out with IBM SPSS Statistics (version 27; IBM Deutschland Ltd., Ehningen, Germany). Normal distribution of all data was verified. The Student *t*-test, chi square, ANOVA variance and multivariate analysis were used to determine influencing factors regarding complications and mortality; 95% confidence intervals and standard deviations were calculated. For data without normal distribution, the Wilcoxon rank test was used. We used the Fisher exact test for description of significant differences in mortality between the groups. Survival analysis was shown with Kaplan–Meier curves. The significance level was set at 5% (*α* = 0.05).

## Results

1933 patients were included and predominantly female (68.9% female and 31.1% male). The average age was 79.8 years (range: 18–103; SD 12). Only 119 patients were younger than 60 years (6.2%). The mean BMI was 24.38 kg/m^2^ (range: 11.7–66 kg/m^2^). The patients were divided into 867 femoral neck fractures, 928 pertrochanteric and 138 subtrochanteric fractures. In 385 cases, total hip endoprothesis was implanted. Four hundred forty-six patients received a hemiarthroplasty. Cephalomedullary nailing was done in 1055 cases, 40 patients obtained DHS and seven screw or plate osteosynthesis. There was no difference of BMI or gender distribution between the fracture types. In 81 cases, a concomitant fracture was also treated surgically within the same hospital admission.

### Pre-operative status

Information about care level was available for most patients. 1157 patients (59.9%) had been self-sustaining without caretaking whilst 776 (40.1%) already had caretaking prior to hospital admission (level 1: 140, level 2: 289, level 3: 196; level 4: 138, level 5: 13). Pre-operative mobility was already reduced in 53.8% of the cohort.

**Fig. 1:**
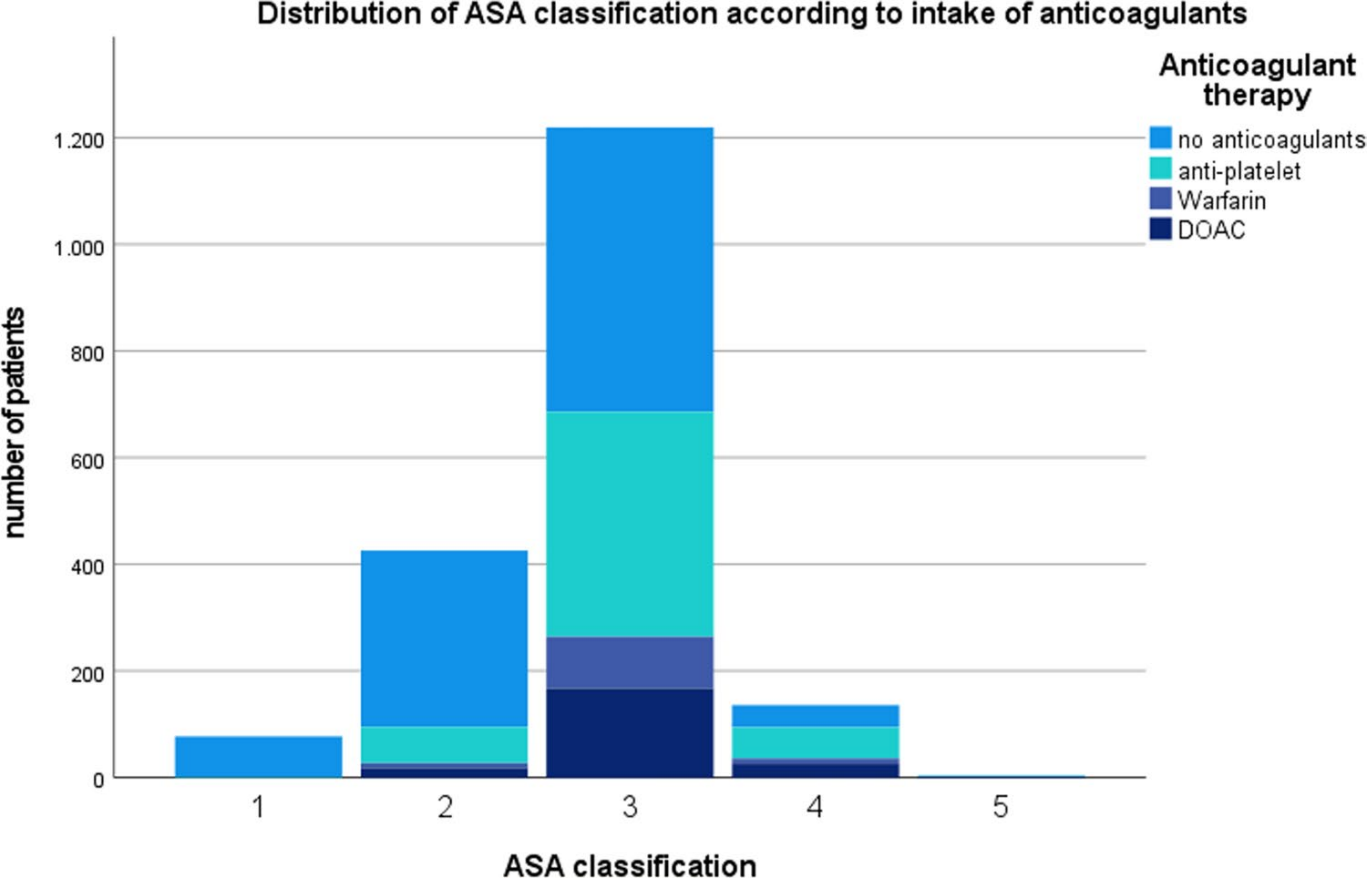
Distribution of ASA classification according to intake of anticoagulants

The average CCI for the total cohort was 5.87 points (range: 0–15, SD 2.455). Patients without anticoagulant therapy had a significantly lower index at 5.02 points (SD 2.46). Amongst all the other patients, no relevant difference was recorded (anti-platelet: 6.73; Warfarin: 6.77, DOAC: 7.04 points). The ASA classification can be seen in Fig. 1. Patients who died post-operatively retrospectively had a higher CCI than the survival group (7.56 vs. 5.81; range: 0–15, SD 2.4; *p* < 0.000).

### Time to surgery

On average, surgery could be performed after 25.9 h (range: 0.95–256.6, SD 20.2 h) after hospital admission. 62.9% could be treated within 24 h, another 25.8% met the 48-h time limit and 11.3% had a delayed surgery for more than 48 h. Younger patients had a significantly shorter wait (*p* < 0.000). Reasons for delay were anticoagulant therapy according to our treatment algorithm, comorbidities, infection at time of admission and cardiovascular risk factors as well as operating room (OR) or intensive care unit (ICU) capacity. Trochanteric fractures were admitted to OR quicker than femoral neck fractures (24.5 h vs 28.4 h; *p* < 0.009).

### Length of hospital stay

The mean LHS was 13.7 days (SD 7.2 days). Four hundred sixty-five (24%) patients were treated on ICU post-operatively for an average of 0.52 days (range: 0–23). LHS was prolonged by the occurrence of complications as well as patient age (*p* < 0.001) and type of operation (longer stay for arthroplasty than for osteosynthesis).

### Anticoagulant therapy

**Fig. 2:**
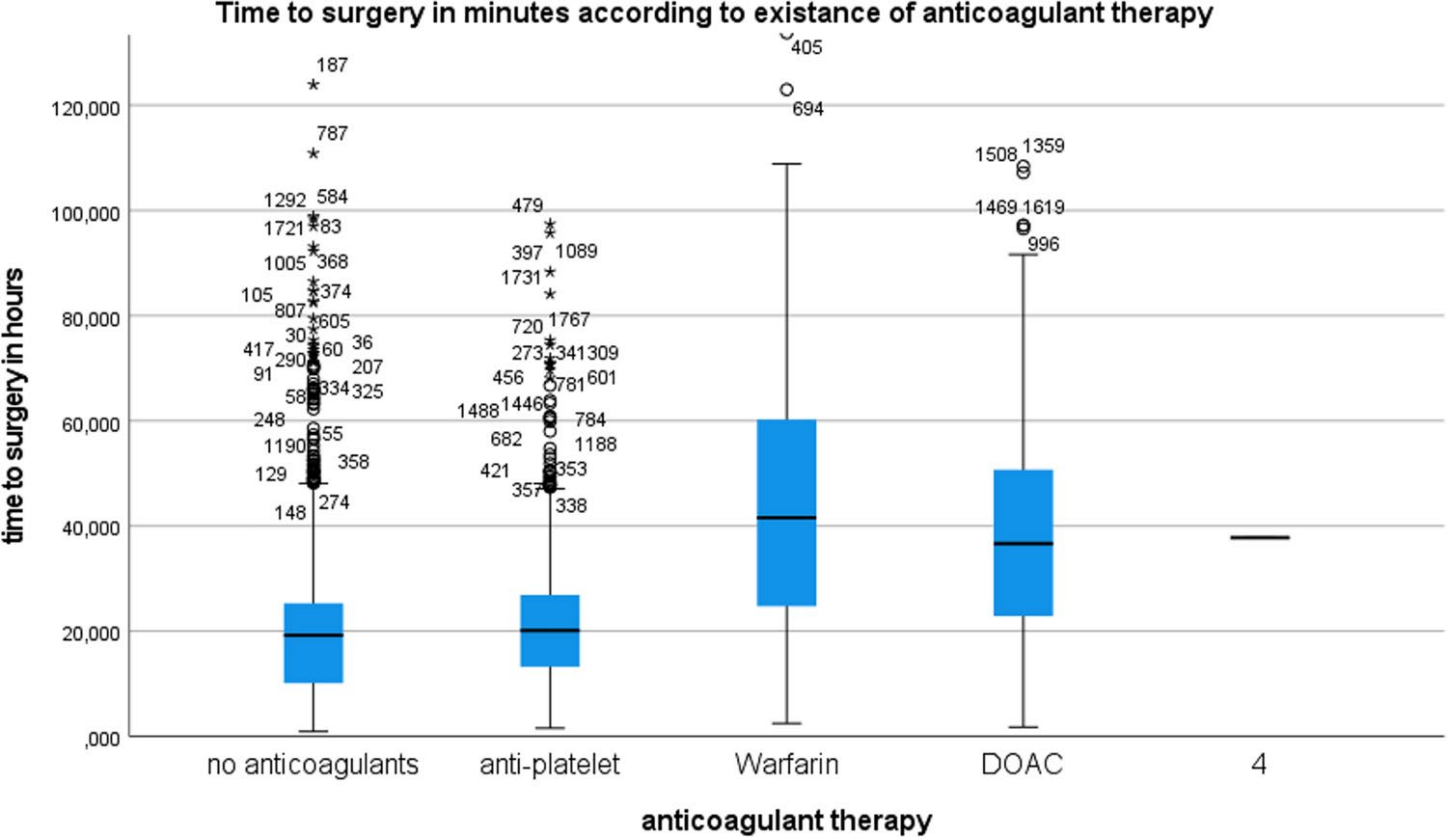
Time to surgery in minutes according to existence of anticoagulant therapy

Three hundred fifty-one patients (17.9%) were on anticoagulant therapy when administered and 585 patients had anti-platelet therapy (29.9%). Divided into subgroups, 118 were on warfarin and 222 on DOACs. 52.2% of the cohort had no anticoagulants. Figure 2 shows patients with anticoagulants having had a delay to surgery of 41.37 h vs 22.1 h for patients without and 23.5 h for patients with anti-platelet therapy (*p* < 0.000). Patients with anticoagulants spent a significant longer time on intensive care post-operatively (*p* < 0.002). A difference between patients on warfarin and DOACs could not be seen. The existence of anticoagulants showed a significant correlation to the occurrence of complications specifically pneumonia, urinary tract infection and wound infection (*p* < 0.013).

### Complications

The total complication rate was 22.4%. Surgical site complications consisted of infections with necessity of revision surgery and i.v. antibiotics, re-fractures, cutting out and dislocations after arthroplasty. In case of infection, the most common pathogen was *Staphylococcus aureus*. On average every patient with infection required two revisions including debridement, antibiotic therapy, and implant retention “DAIR”. Haematoma was noted in 58 cases but did not need surgical revision. Haematoma did occur more often in patients with anticoagulants.

Urinary infections occurred in 193 patients (9.9%) and were treated with antibiotics. Pneumonia was seen in 111 patients (x-ray, labs and symptoms) and also treated with i.v. antibiotics and mobilisation.

There was no difference in the complication rate between the surgical procedures. Patients with complications retrospectively showed a prolonged time to surgery in comparison to those without complications (28.9 h vs 24.9 h; *p* < 0.00). The length of hospital stay was substantially longer in case of complications (17.37 vs. 12.5 days; *p* < 000). Patients with complications were significantly older (79.1 vs. 82.1 years; *p* < 0.00).

### Mortality

**Fig. 3:**
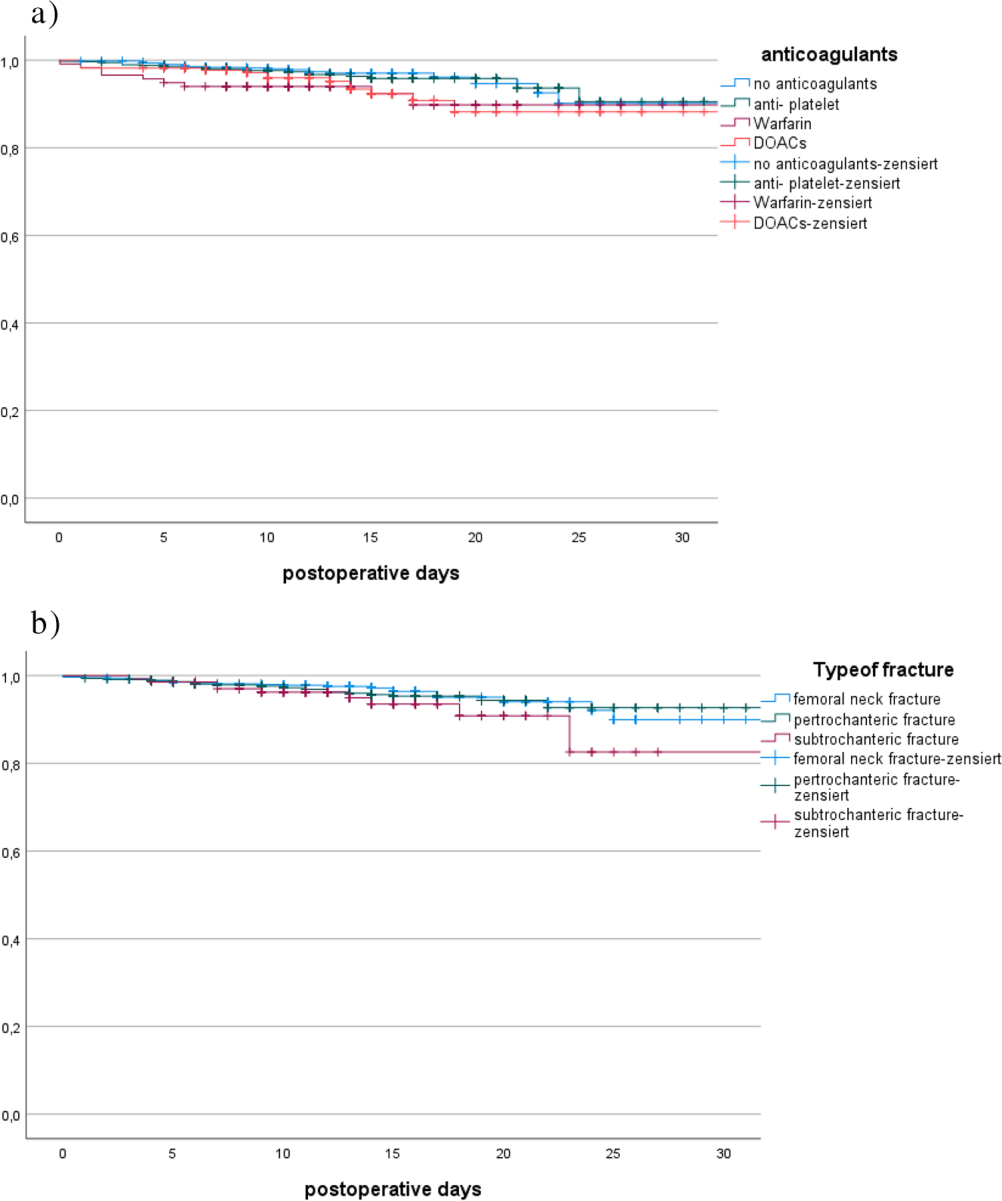
Kaplan–Meier survival rates. **a** Survival by anticoagulants. **b** Survival rate by fracture type

The reasons for in-house mortality were lung embolism, cardiac arrest, pneumonia including aspiration and sepsis. The overall in-house mortality rate was 4% (*N* = 78). Only 3% (*N* = 31) of the patients without anticoagulants died and 3.4% of the patients with anti-platelet therapy. In contrast to these findings, the group with either DOACs or warfarin showed a nearly twice as high death rate of 7.7% (*N* = 27), *p* < 0.00. Again, the impact of time to surgery is noticeable as the delay was much longer in the group of deceased patients in comparison to the survival group (37.5 vs. 25.5 h; *p* < 0.000). Whilst there was no significant difference in the mortality rate between the groups with surgery within 24 and 48 h (2.9% vs. 3.8%; *p* < 0.535), there was a significant increase in mortality of patients who had to wait for surgery for more than 48 h (9.8%; *p* < 0.001) (Fig. 3).

### Multivariate analysis—Cox regression

The multivariate analysis reveals definitive influential factors with regard to the survival rate after surgery for proximal femur fractures (Table 1). Age, gender and BMI have a significant influence on mortality rates (adjusted hazard ratio age: 1.082; gender: 1.775; BMI: 0.909). Amongst the fracture morphology subtrochanteric fractures have a higher likelihood of mortality (HR: 2.504; CI:1.154–5.436). Furthermore, the prevalence of comorbidities (CCI) and the occurrence of complications affect survival rates. Within patients on anticoagulants, the existence of DOACs impacts the mortality rate most (HR:1.913; CI: 0.979–3.739).

**Table 1:** Multivariate analysis (Cox regression) on mortality after proximal femur fractures

Parameter	Significance *p* value	HR (95% confidence interval)
Fracture type (reference: femoral neck fracture)	0.61	
*Pertrochanteric fracture*	0.668	1.120 (0.667–1.879)
*Subtrochanteric fracture*	0.020	2.504 (1.154–5.436)
Age	< 0.001	1.082 (1.048–1.116)
Gender	0.031	1.775 (1.055–2.987)
Complications	< 0.001	2.370 (1.436–3.909)
BMI	0.004	0.909 (0.852–0.971)
ICU stay	0.006	1.993 (1.224–3.244)
Anticoagulant therapy (reference: no anticoagulants)	0.336	
*Anti-platelet*	0.769	1.094 (0.605–1.978)
*Warfarin*	0.210	1.653 (0.753–3.627)
*DOAC*	0.059	1.913 (0.979–3.739)
Charlson Comorbidity Index	0.001	1.2110 (1.081–1.354)

## Discussion

Various studies have been conducted to detect the most important factors influencing the outcome and complication rates after hip fractures. Many have agreed on the vital importance of early surgery for all patients, independent of individual risk factors and comorbidities [14–20].

Our aim was to give more attention to the individual increasing patient risk factors especially anticoagulant therapy and take into consideration the pre-operative patients’ status than merely the time to surgery. Therefore, we established a therapy algorithm for patients with anticoagulants based on renal (dys-) function, risk level of surgery and type of anticoagulants. Trying to stay within a 24- or 48-h limit is challenging for every clinical setting as OR capacity itself may be a limiting factor. Patients with comorbidities and especially anticoagulant therapy are particularly challenging. By prolonging the delay to surgery to a maximum of 48 h for patients on anticoagulants and limited renal clearance time is gained to optimise patient comorbidities and pre-operative status without increasing the mortality rate.

Eighty-nine percent of our patients could be treated within 48 h after admission but the overall patients on anticoagulants showed an increased delay of time to surgery. The overall complication rate was 22.4% and was therefore compatible to complication rates described in previous studies [14, 21]. The in-house mortality rate was 4% but showed a significant increase for patients on anticoagulants (7.7%) consistent with other studies showing increased mortality for anticoagulants [22]

Dettoni et al. were able to determine two main factors for increased mortality and complications, namely warfarin and a delay in time to surgery in a large cohort of 875 hip fractures [23]. DOACS were evaluated as a risk for higher mortality and delay to surgery also by a large metaanalysis [24]. Protocols for patients with anticoagulants have been established to enable early surgery [25].

In contrast to these findings, Caruso et al. compared warfarin medication to anti-platelet therapy and a control group and demonstrated a higher but not significant difference of mortality rate in the warfarin group and, more importantly, could not show any influence of time to surgery for mortality rates [26]. We did not find different mortality rates amongst the various types of anticoagulants (warfarin and DOACs).

The question remains whether the determining factor is mainly time to surgery, if anticoagulants play a substantial role themselves or if they only lead to a higher mortality rate by prolonging the wait for surgery. The latter is supported by a study by Mattisson et al. who compared two groups of patients with trochanteric fractures with and without warfarin which were both treated within 24 h after supplementing Vit K and PCC [27]. They could not find an increased risk for post-operative complications or a significant change of mortality rate. A small study comparing patients with and without DOACs concluded that outcome results were similar and delaying surgery was not effective [28].

The evaluation of comorbidities showed that the patients with anticoagulant therapy had more comorbidities than patients without. Therefore, mortality increase for patients with anticoagulants may be biased. If anticoagulants were present, there was no significant difference whether the medication was simply anti-platelet medication, DOAC or warfarin. The bias is increased by the fact that all the younger patients (< 55 years) included for a femoral neck fracture required urgent surgery (head-preserving) within 6 h. The majority of those patients also had fewer comorbidities and were classified ASA I/II.

In agreement to our data, age has been proven by many to be a risk factor after hip fractures for poor outcome. Delgrado et al. found cognitive impairment to have a vital influence on the outcome after hip fractures. They also concluded that dementia was a risk factor for post-operative complications for example pneumonia und urinary tract infection [29]. Our multivariate analysis has also shown the influence of independent factors, such as age, on mortality rates. In contrast to the concept of paradox obesity, we found mortality increased with higher body mass index [30].

Studies have shown that comorbidities are a valid assessment tool and especially the Charlson Comorbidity Index more reliable in predicting the outcome than the ASA classification [31]. Fröhlich et al. claim that comorbidities and age over 75 years are associated with a higher mortality up to more than a year [32]. A further large study showed CCI to be the dominant factor influencing of short- and long-term mortality after hip fractures [33].

The one variable which can be primarily influenced during the hospital stay is the critical time to surgery, which also had a significant influence on mortality and on the occurrence of complications. But our data also shows that if surgery is performed within 48 h of admission there is no significant increase of mortality whereas death rates rise significantly for patients waiting for more than 48 h until surgery. We conclude that, if necessary, this time window of 48 h can be used to optimise the patients’ pre-operative status without increasing risks.

The limitations of this study are the retrospective design and the lack of long-term data, such as a one year mortality rate. Randomised control studies would be preferable but difficult to establish based on current knowledge.

## Conclusion

Preexisting anticoagulant therapy and comorbidities in patients with proximal femur fractures is associated with a higher mortality rate, risk of complications and prolonged hospital stay. Further influential factors are age, gender, BMI and time to surgery. Patients treated within 48 h do not have an increased mortality risk. So a 48-h window of opportunity can be used to optimise patients’ pre-operative status and give enough time to clear anticoagulant therapy.

## Data Availability

All data concerning the study is available.
